# Supplementary figures and images for: Association of genetic variation in COL11A1 with adolescent idiopathic scoliosis
Source: eLife. 2024 Jan 26;12:RP89762. doi: 10.7554/eLife.89762 (PMC10945706; doi:10.7554/eLife.89762)

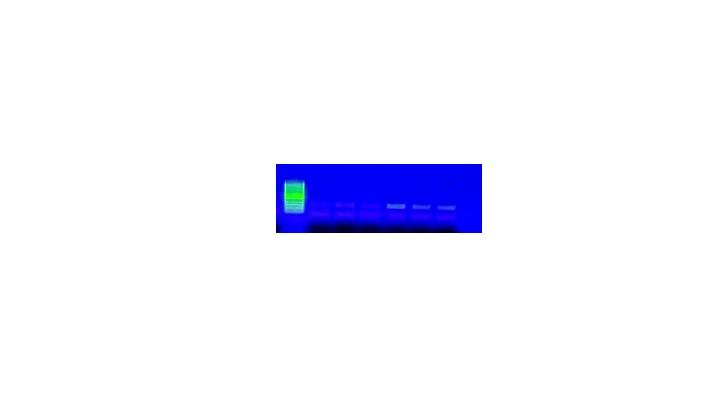

Supplement: Figure 4—source data 1. [file elife-89762-fig4-data1.zip › Figure 4 XXX Source data 1.jpg]

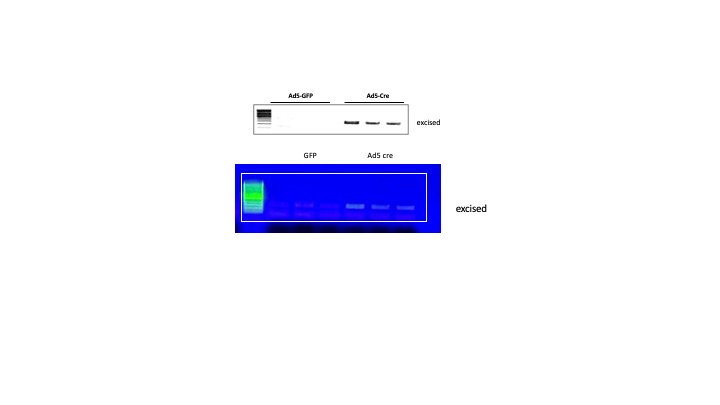

Supplement: Figure 4—source data 2. [file elife-89762-fig4-data2.zip › Figure 4 XXX Source data 2.jpg]

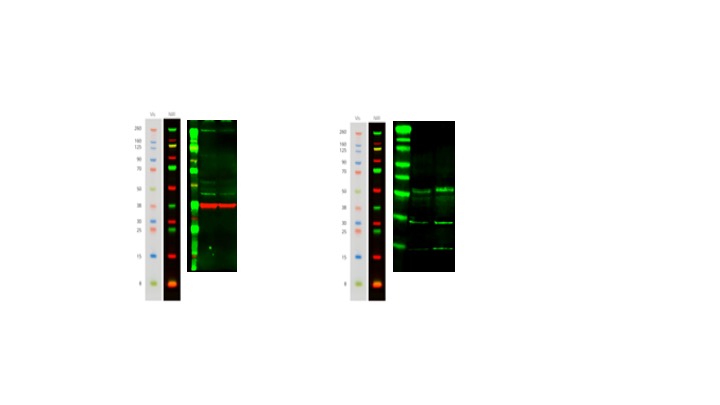

Supplement: Figure 4—source data 3. [file elife-89762-fig4-data3.zip › Figure 4 XXX Source data 3.jpg]

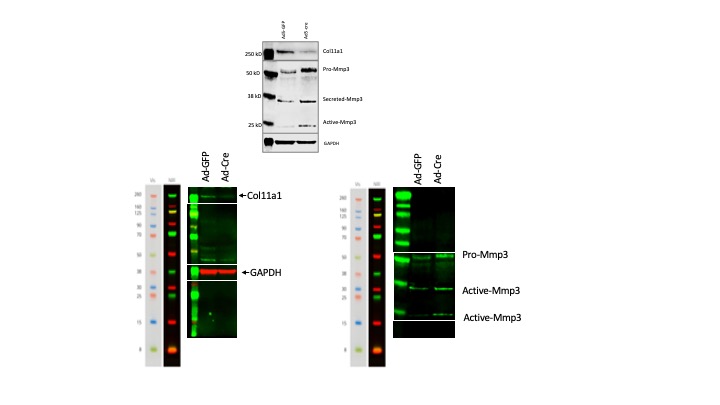

Supplement: Figure 4—source data 4. [file elife-89762-fig4-data4.zip › Figure 4 XXX Source data 4. .jpg]

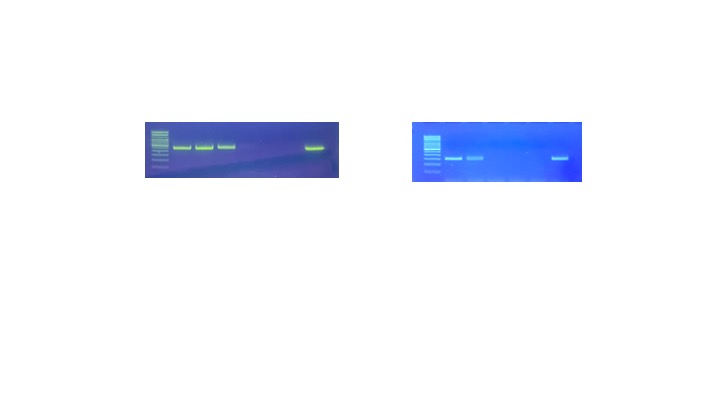

Supplement: Figure 4—figure supplement 3—source data 1. [file elife-89762-fig4-figsupp3-data1.zip › Figure 4 XXX Figure supplement 3 Source data 1. .jpg]

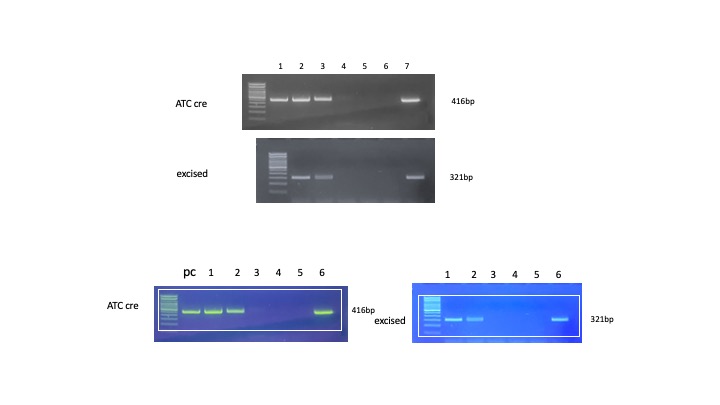

Supplement: Figure 4—figure supplement 3—source data 2. [file elife-89762-fig4-figsupp3-data2.zip › Figure 4 Figure supplement 3 Source data 2. .jpg]

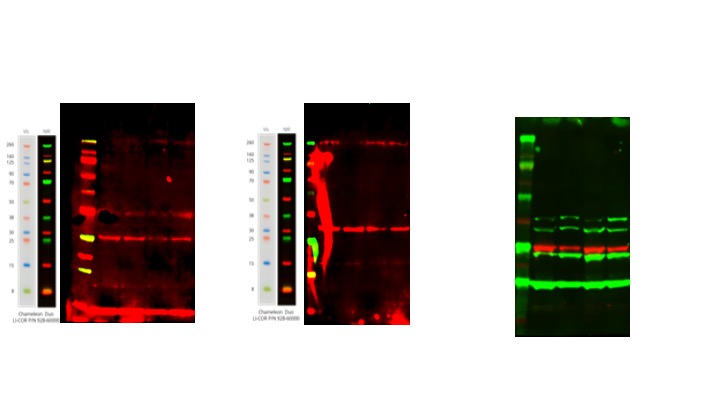

Supplement: Figure 5—source data 1. [file elife-89762-fig5-data1.zip › Figure 5 Source data 1. .jpg]

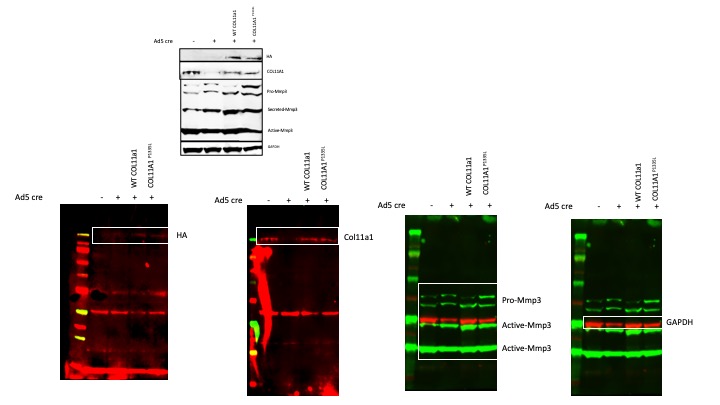

Supplement: Figure 5—source data 2. [file elife-89762-fig5-data2.zip › Figure 5 Source data 2.jpg]

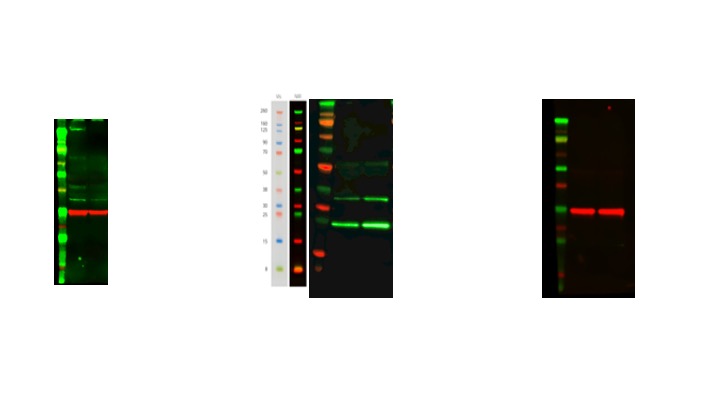

Supplement: Figure 6—source data 1. [file elife-89762-fig6-data1.zip › Figure 6 XXX Source data 1.jpg]

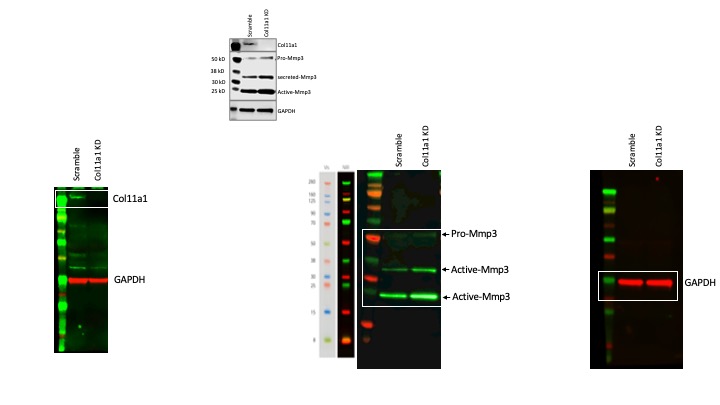

Supplement: Figure 6—source data 2. [file elife-89762-fig6-data2.zip › Figure 6 XXX Source data 2.jpg]
